# Supplementary figures and images for: A pilot study of inflammatory mediators in brain extracellular fluid in paediatric TBM
Source: PLoS One. 2021 Mar 12;16(3):e0246997. doi: 10.1371/journal.pone.0246997 (PMC7954352; doi:10.1371/journal.pone.0246997)

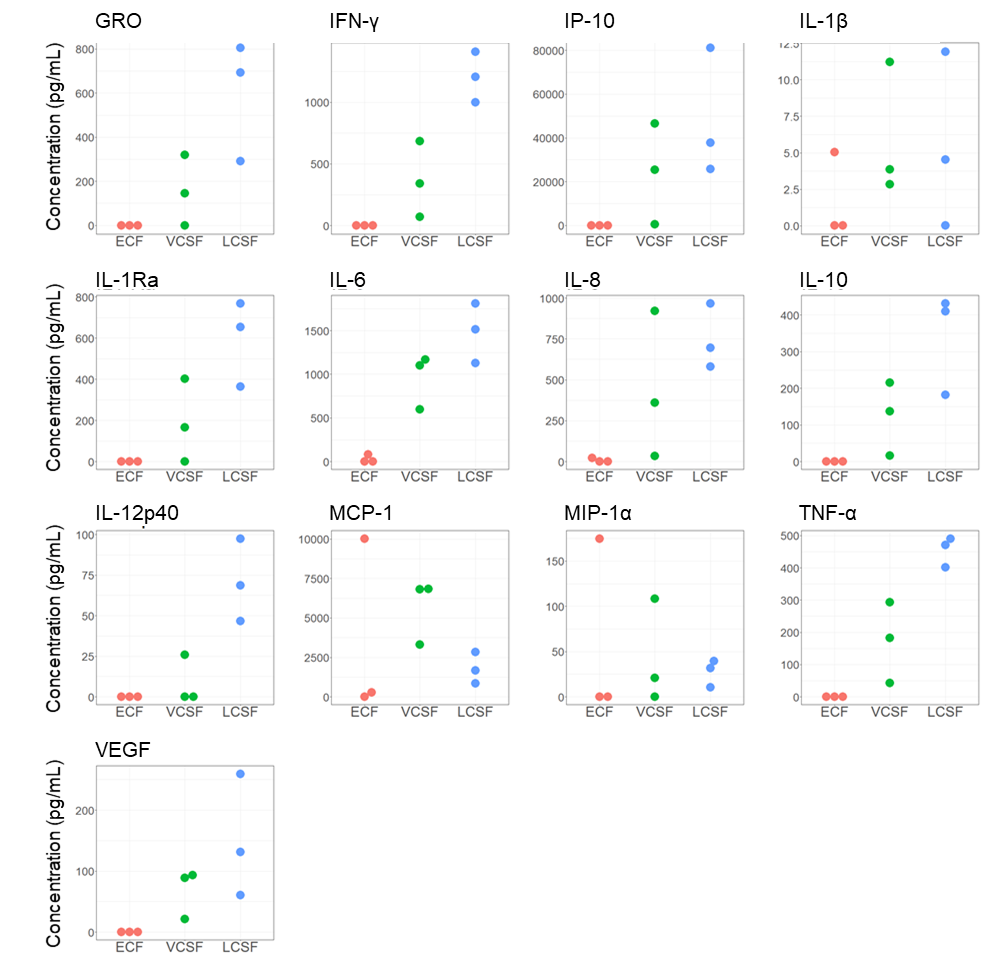

Supplement: S1 Fig — Each LCSF sample analysed (n = 3) is plotted with its paired ECF and VCSF which were obtained from the same patient at the same time point. Plotted values have been shifted horizontally to avoid overplotting. Abbreviations: ECF, extracellular fluid; IFN-γ, interferon γ; IL, interleukin; IL-1Ra, interleukin 1 receptor antagonist; IP-10, interferon-γ inducible protein-10; LCSF, lumbar cerebrospinal fluid; MCP-1, monocyte chemoattractant protein; MIP-1α, macrophage inflammatory protein 1α; TNF-α, tumour necrosis factor-α; VEGF, vascular endothelial growth factor; VCSF, ventricular cerebrospinal fluid. (TIF) [file pone.0246997.s001.tif]

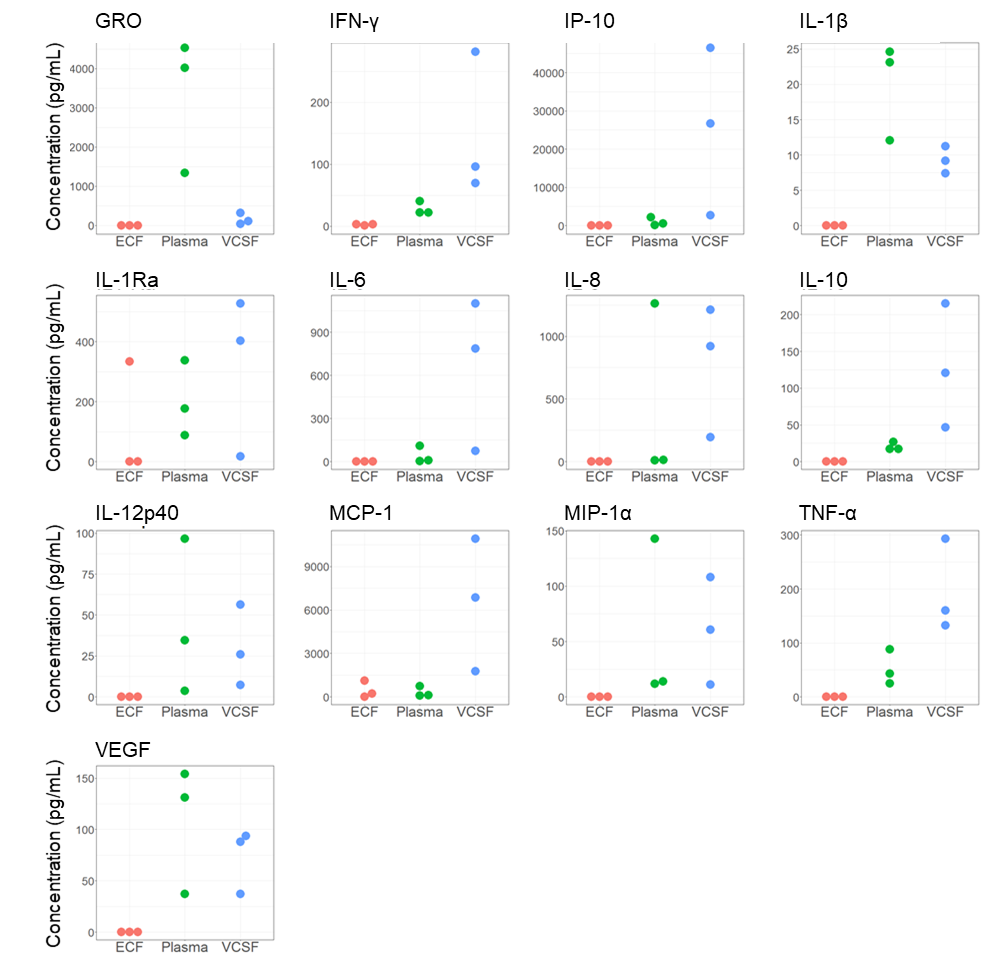

Supplement: S2 Fig — Each plasma sample analysed had both a paired ECF and VCSF which were obtained from the same patient at the same time point (n = 3 cases). Plotted values have been shifted horizontally to avoid overplotting. Abbreviations: ECF, extracellular fluid; IFN-γ, interferon γ; IL, interleukin; IL-1Ra, interleukin 1 receptor antagonist; IP-10, interferon-γ inducible protein-10; LCSF, lumbar cerebrospinal fluid; MCP-1, monocyte chemoattractant protein; MIP-1α, macrophage inflammatory protein 1α; TNF-α, tumour necrosis factor-α; VEGF, vascular endothelial growth factor; VCSF, ventricular cerebrospinal fluid. (TIF) [file pone.0246997.s002.tif]
