# Supplementary material for: A pilot study of inflammatory mediators in brain extracellular fluid in paediatric TBM
Source: PLoS One. 2021 Mar 12;16(3):e0246997. doi: 10.1371/journal.pone.0246997 (PMC7954352; doi:10.1371/journal.pone.0246997)
Supplement: S1 Table — (DOCX) [file pone.0246997.s003.docx]

| Cytokine | Median ECF concentration (pg/mL) | Median Ventricular CSF Concentration (pg/mL) | ECF to Ventricular CSF ratio (%) |
| --- | --- | --- | --- |
| GRO | 0.01 | 37.37 | 0.03 |
| IFN-γ | 1.92 | 300.5 | 0.64 |
| IL-10 | 0.01 | 54.8 | 0.02 |
| IL-12p40 | 0.01 | 7.26 | 0.14 |
| IL-1β | 0.01 | 4.53 | 0.22 |
| IL-1Ra | 0.01 | 166.99 | 0.01 |
| IL-6 | 0.01 | 785.46 | 0.00 |
| IL-8 | 0.01 | 194.07 | 0.01 |
| IP-10 | 30.67 | 13592.84 | 0.23 |
| MCP-1 | 873.78 | 6814.58 | 12.82 |
| MIP-1α | 0.01 | 11.08 | 0.09 |
| TNF-α | 0.01 | 132.46 | 0.01 |
| VEGF | 0.01 | 63.19 | 0.02 |
| Medians calculated using the 9 pairs of time linked ECF and Ventricular CSV samples. 0.01pg/mL was the value assigned to concentrations below the lower detection limit of detection of the test kit. Abbreviations: ECF, extracellular fluid; CSF, cerebrospinal fluid; IFN-γ, interferon-γ; IL, interleukin; IL-1Ra, interleukin 1 receptor antagonist; IP-10, interferon-γ inducible protein 10; MCP-1, monocyte chemoattractant protein; MIP-1α, macrophage inflammatory protein 1α; TNF-α, tumour necrosis factor-α; VEGF, vascular endothelial growth factor. | | | |

**S1 Table. Paired brain extracellular fluid and ventricular cerebrospinal fluid cytokine concentrations**
