# Supplementary material for: A pilot study of inflammatory mediators in brain extracellular fluid in paediatric TBM
Source: PLoS One. 2021 Mar 12;16(3):e0246997. doi: 10.1371/journal.pone.0246997 (PMC7954352; doi:10.1371/journal.pone.0246997)
Supplement: S3 Table — (DOCX) [file pone.0246997.s005.docx]

**S3 Table. Metabolite differences between radiological outcome and mortality**

| Metabolite | Lactate-pyruvate ratio | Glycerol (pg/mL) | Glutamate (pg/mL) | Glucose (pg/mL) |
| --- | --- | --- | --- | --- |
| Group A | 19.51 | 40.28 | 1.01 | 1.19 |
| Group B | 24.24 | 145.79 | 1.03 | 0.61 |
|  |  |  |  |  |
| Survived | 19.23 | 24.62 | 0.90 | 1.14 |
| Died | 23.43 | 76.03 | 1.13 | 0.93 |
| Median cytokine concentrations for patients with focal or no infarcts (Group A) relative to patients with bilateral or extensive infarcts (Group B), and between patients who survived and those who died. Abbreviations: IL, interleukin; IL-1Ra, interleukin 1 receptor antagonist; MCP-1, monocyte chemoattractant protein | | | | |
